# Defibrillation in children

**DOI:** 10.4103/0974-2700.66526

**Published:** 2010

**Authors:** Sarah E Haskell, Dianne L Atkins

**Affiliations:** Department of Pediatrics, University of Iowa Children’s Hospital, University of Iowa Carver College of Medicine, 200 Hawks Drive, Iowa City, IA 52242, USA

**Keywords:** Defibrillation, pediatrics, ventricular fibrillation

## Abstract

Defibrillation is the only effective treatment for ventricular fibrillation (VF). Optimal methods for defibrillation in children are derived and extrapolated from adult data. VF occurs as the initial rhythm in 8-20% of pediatric cardiac arrests. This has fostered a new interest in determining the optimal technique for pediatric defibrillation. This review will provide a brief background of the history of defibrillation and a review of the current literature on pediatric defibrillation. The literature search was performed through PubMed, using the MeSH headings of cardiopulmonary resuscitation, defibrillation and electric countershock. The authors’ personal bibliographic files were also searched. Only published articles were chosen. The recommended energy dose has been 2 J/kg for 30 years, but recent reports may indicate that higher dosages may be more effective and safe. In 2005, the European Resuscitation Council recommended 4 J/kg as the initial dose, without escalation for subsequent shocks. Automated external defibrillators are increasingly used for pediatric cardiac arrest, and available reports indicate high success rates. Additional research on pediatric defibrillation is critical in order to be able to provide an equivalent standard of care for children in cardiac arrest and improve outcomes.

## INTRODUCTION

Defibrillation is the delivery of an electric shock during cardiac arrest for ventricular fibrillation (VF) or ventricular tachycardia (VT). VF occurs in 8–20% of pediatric cardiac arrests.[1–5] For every 1 min delay in defibrillation, the survival rates fall by 7–10%.[6] Although defibrillators were first described in the mid-1900s and have been used extensively for children in cardiac arrest, unanswered questions still persist about their use in children. The optimal technique and energy dosing for pediatric defibrillation have generally been derived from extrapolation from adult clinical data or small pediatric studies. For this reason, the optimal energy dose to successfully defibrillate a child is unknown and is the most important question to be answered. Discussion regarding the most appropriate pad or paddle size, placement of pads or paddles and use of biphasic waveforms also exists. Research to answer these questions has been slow because the frequency of cardiac arrest in children, especially when due to VF, is much lower than in adults. However, this has improved in recent years as we strive to improve the results of resuscitation in children.

The literature search was performed through PubMed, using the MeSH headings of cardiopulmonary resuscitation, defibrillation and electric countershock. The authors’ personal bibliographic files were also searched. Only published articles were chosen.

## BACKGROUND

### History of defibrillation

A brief history of defibrillation has recently been provided by Acosta *et al*.[7] Although William Kouwenhoven is generally recognized as the developer of the first defibrillator in the early 20^th^ century, he and his team based their groundbreaking research on the work of Prevost and Battelle, published in 1899. Prevost and Battelle had demonstrated that VF could be induced by a weak alternating current that could then be reversed by a higher intensity current. They also observed that “countershock” was not effective if VF persisted for more than 2 min. From these studies, Kouwenhoven and his colleagues developed the initial alternating current internal defibrillator.[7] In 1947, Claude Beck was the first to use open chest defibrillation in humans and, shortly thereafter, Zoll used the first external defibrillator in humans on a closed chest.[8] Interestingly, Beck’s open chest defibrillation occurred in a 14-year-old boy undergoing thoracic surgery![9] Lown *et al*. demonstrated the marked advantages of direct current over alternating current, and quickly demonstrated the usefulness of synchronization.[10,11] The introduction of the direct current defibrillators with a synchronizing circuit capable of discharging during the absolute refractory period of the cardiac cycle has proven to be a safer alternative than alternating current defibrillators. Since then, defibrillators have become a mainstay of cardiac arrest treatment. With increasing sophistication, including variable energy dosing, rhythm identification and improved waveforms, their use has extended to out-of-hospital providers.[12]

### Energy and current flow

Adequate current flow through the heart is required for successful defibrillation. Current is defined as potential divided by resistance (Amps = Volts/Ohms), whereas energy is defined as power multiplied by time (Joules = Watts X Seconds). There is no parallel relationship between energy and current. The current delivered to the myocardium with a given energy is dependent on the transthoracic impedance, which can vary widely among patients. Thus, the same energy dose can potentially deliver varying current to a patient. Additionally, the percentage of current shunted through the thorax, away from the myocardium, influences the net current a patient receives. Transcardiac current fraction (Fc) is the ratio of transcardiac threshold current (Ic) to transthoracic defibrillator threshold current (It). Although the experimental or theoretical Fc was described in a wide range, from 3 to 45%, Lerman found the Fc to be 1–10% in humans, with an average of 4%.[13] Multiple factors influence the actual amount of current delivered. Transthoracic impedance is a function of tissue properties, electrode surface area, pressure on the electrode and body size. Transcardiac fraction (Fc) is affected by clinical conditions of the patient, such as pleural effusions, pneumothorax and chest configuration. Accurate measurement of the current is difficult and cannot be performed in humans.[13,14] Thus, determining optimal current for successful defibrillation is complex.

### Mechanisms of defibrillation

Several theories have evolved to explain the mechanisms of defibrillation. One of the first theories suggested the critical mass theory: defibrillation was successful only when a critical mass of myocardium was made inexcitable.[15,16] Excitable cells are simultaneously depolarized, thereby extinguishing activation of waveforms within the critical mass of the myocardium, but not in all cells. The second theory, theory of vulnerability, was first described by Chen *et al*.[17] They proposed that defibrillation is mediated not only by depolarizing fully excitable cells but also by depolarizing cells in the relative refractory period. This is achieved by delivering a stimulus that exceeds the upper limit of vulnerability to all regions of the myocardium.

### Defibrillator waveforms

Until the 1990s, monophasic waveforms were used exclusively in defibrillators. Monophasic defibrillators deliver current in one direction whereas biphasic defibrillators deliver current in two directions (positive and negative). For biphasic defibrillators, the current flows in one direction during the first phase of the waveform and in the opposite direction during the second phase. Monophasic defibrillators are typically used according to a protocol of escalating shock energies. In contrast, biphasic waveforms deliver lower energy levels and do not all use escalating energy protocols. Biphasic waveforms have increased the defibrillation efficacy, return of spontaneous circulation (ROSC) and survival to hospital admission, but, as yet, have not been shown to improve hospital discharge[18–20] In a multicenter, randomized, controlled trial for out-of-hospital cardiac arrest, Schneider *et al*. showed that a higher percentage of patients achieved ROSC after 150 J biphasic waveform defibrillation compared with higher energy monophasic waveform defibrillation. Rates of survival to hospital admission and to hospital discharge did not differ between the groups.[18] In a retrospective analysis of out-of-hospital cardiac arrest with VF as initial rhythm from November 1990 to December 2006, Hess *et al*. observed an increase in the sustained ROSC with defibrillation shocks alone after the transition to biphasic waveform defibrillators from monophasic defibrillators. There was a trend toward increased hospital discharge, but it was not statistically significant. Conversely, Kudenchuk *et al*. found no statistically significant difference between waveforms in the likelihood of terminating VF, restoring an organized rhythm or circulation, improving hospital admission or survival after cardiac arrest in a randomized pre-hospital trial comparing monophasic waveforms with biphasic waveform.[21]

To date, no study has compared monophasic and biphasic waveforms in children, but several animal studies demonstrate that similar defibrillation efficacy exists. Using a porcine model of pediatric defibrillation with “infant” and “child” piglets, Clark *et al*. demonstrated the superiority of biphasic waveforms compared to monophasic waveforms.[22] In this study of short duration VF, the 10 ms biphasic waveform was superior to the 5 ms monophasic waveform in the “infant” group at 10, 20, and 30 J energies and at 20 and 30 J energies in the “child” group. Similar findings were obtained when comparing the 10 ms monophasic waveform to the 10 ms biphasic waveform. Two studies demonstrated that the escalating “pediatric” biphasic dosage strategy was at least as safe and effective as the standard weight-based monophasic dose over a wide range of weights in a piglet model of prolonged pre-hospital VF.[23,24] With the proposed superiority of biphasic waveforms, manufacturers are implementing biphasic waveforms in their defibrillators and have discontinued the production of monophasic models. However, Kudenchuk *et al*. reported that an estimated 500,000 monophasic waveform defibrillators are still in clinical use.[21] Even with a possible superior waveform, both monophasic and biphasic waveforms are successful in terminating VF, and their use is critical in early defibrillation for out-of-hospital cardiac arrests.

## PEDIATRIC DEFIBRILLATION

### Energy dose

**Figure 1:**
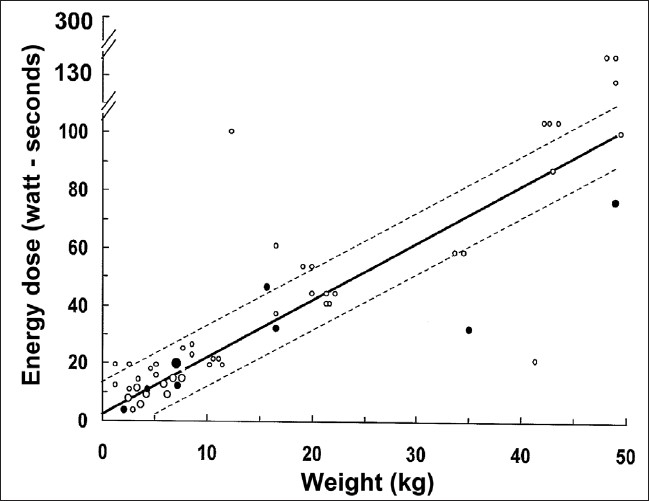
Energy dose versus weight. The solid line represents a dose of 2 J/kg. Dotted lines represent 2 J/kg ± 10 J. Closed circles represent unsuccssful shocks and open circles represent successful shocks, defined as termination of ventericular fibrillation. Size of the circle represents the number of patients (modified from Gutgesell, 1976)

The American Heart Association (AHA) currently recommends 2 J/kg, escalating to 4 J/kg, in pediatric patients. These recommendations are based on extrapolated animal data,[25] adult data[26,27] and one small retrospective pediatric study.[28] Gutgesell reported 27 patients with in-hospital cardiac arrest, with the primary endpoint of termination of defibrillation. The numbers of shocks, survival, hospital discharge or neurologic outcomes were not reported. All shocks were delivered with monophasic waveforms. Although they concluded that 2 J/kg is an effective dose, the range of doses that patients received was probably quite broad, especially for the younger and smaller patients. Their analysis used 2 J/kg ± 10 J as the dose range. This resulted in a much broader range of dosing for infants compared to adolescents, confusing the issue of dosing [Figure 1].

Because of the absence of any additional data in children, no changes have been made to the AHA guidelines for defibrillation. Those guidelines are based on recommendations established 30 years ago. With the recognition that VF occurs as the initial rhythm in 10% of the children[2,4,5] and occurs in approximately 25% at some point during the resuscitation, there has been increasing interest in establishing an optimal energy dose for children. The use of automated external defibrillators (AEDs) in children and biphasic waveform has also prompted a review of appropriate dosing.[29] In Clark’s piglet study, a single shock of 2 J/kg was successful <10% of the time.[22] With biphasic waveform, 2 J/kg yielded only 25% success rates for “infant” piglets and 32% for “child” piglets. In marked contrast, high success rates (>80%) were achieved by using 4 J/kg biphasic waveforms for “infant” piglets and 3 J/kg for “child” piglets for short duration VF. In 2005, Berg[30] retrospectively reviewed cardiac arrests in children <13 years of age over a 5-year period, attempting to replicate the Gutgesell study,[28] but for out-of-hospital arrest. An energy dose of 2 J/kg ± 10 J terminated prolonged VF in only 50% of the children. However, most were converted to asystole or pulseless electrical activity. None survived to hospital discharge. Rodriguez-Nunez *et al*. reported that only 18% of the children defibrillated at 2 J/kg terminated VF on first shock.[31] Thirty-eight percent of the children required greater than shocks to terminate VF and pulseless VT. They reported that the survival rate was higher in patients treated with a second shock dose of 2 J/kg than those who received higher doses. Rossano *et al*. demonstrated that many children do not actually receive 2 J/kg when resuscitated in the field.[32] In a retrospective review of three Emergency Medical Systems (EMS) systems with primarily monophasic waveforms, children received an average first dose of 3.87 J/kg based on estimated weights. Only 12% received the recommended 2 J/kg. This study reported a very high rate of hospital discharge of 33%. A major drawback in all these studies is that monophasic defibrillators were the primary waveform.

One out-of-hospital study using biphasic waveforms doses, at a fixed pediatric dosage (50 J), regardless of weight, demonstrate a high successful defibrillation rate.[29] Thus, the data suggest that higher, rather than lower, dosages are needed for effective pediatric defibrillation even with the advent of biphasic defibrillation. This is likely because our conclusions from the prior adult and animal studies underestimated the energy needed for defibrillation. Based on this newer data, the European Resuscitation Council (ERC) changed their guidelines in 2005, and recommended a single-shock strategy using a non-escalating dose of 4 J/kg for defibrillation in children.[33]

Recognizing that 2 J/kg is often unsuccessful at defibrillation on the first shock and escalating doses of energy and multiple shocks are often required for successful defibrillation in children, raises the important question of whether there is a safe upper limit and whether there is a toxicity of energy from cumulative doses. In 1980, Babbs *et al*.[34] demonstrated that the median toxic dose for monophasic waveforms, as measured by histopathologic exam, was 30 J/kg in adult animals and the median lethal dose was 470 J/kg. Similarly, Gaba *et al*.[35] did not detect myocardial damage in neonatal piglets, as measured by technetium-99m pyrophosphate scanning, until cumulative doses of 150 J/kg were delivered into the sinus rhythm. More recently, Berg *et al*. compared the safety of monophasic weight-based shocks and “pediatric” shocks with biphasic waveform in a piglet model of long-duration VF.[24,36] “Pediatric” shocks were performed by delivering biphasic doses of approximately 51/71/81 J, and the control piglets received monophasic 2/4/4 J/kg. Myocardial function and damage were assessed by specific hemodynamic parameters, including continuous electrocardiogram (ECG) and contrast angiographic assessment of LV ejection fraction at 1, 2, 3 and 4 h post-shock. Piglets shocked with “pediatric” doses, regardless of weight, had better outcomes than monophasic weight-based doses. Tang *et al*.[37] also demonstrated successful defibrillation using adult AEDs in which the energy-reducing electrodes delivered 50 J shocks to piglets ranging from 3.7 to 25 kg. All piglets were successfully resuscitated with the 50 J shocks without compromise of post-resuscitation myocardial function or survival. In 2008, Berg demonstrated, using a swine model, that the adult dose biphasic defibrillators with shocks of 200, 300 and 360 J compared to “pediatric” dose shocks of 50, 75, and 85 J through attenuated biphasic defibrillators resulted in a greater frequency of myocardial damage and worse post-resuscitation myocardial function.[23,24] However, there are several case reports and one small case series of biphasic fixed-energy dosing where children aged 6 months to 2 years received high doses (>5 J/kg) with successful termination of VF and survival.[29,38–40] Although no specific upper limit for dosing or toxic dose has been defined for children, multiple studies have demonstrated the safe use of “pediatric” doses that are successful regardless of weight, and are well above the currently recommended 2–4 J/kg.

### Escalating dosage

In children, the current AHA guidelines recommend an initial dose of 2 J/kg, and escalating to 4 J/kg if the first one to two shocks are unsuccessful, while the ERC does not recommend escalation beyond the initial dose of 4 J/kg.[33,41] Presently, no recommendations have been made about further escalation of energy doses for pediatric patients requiring multiple shocks. A recent adult study for patients with out-of-hospital cardiac arrest who received greater than one shock compared a fixed, lower energy (150–150–150 J) with escalating, higher energy (200–300–360 J) regimens. In this study, Stiell *et al*.[42] found higher rates of VF conversion and termination with an escalating higher-energy regimen for patients requiring multiple shocks. Even when “pediatric” piglets receive adult energy dosing, not all animals were successfully defibrillated with one shock, and required escalating dosage.[24] It will be difficult to determine the need for escalation until the best initial dose has been established.

### Pediatric pad size and position

There are no studies in children that directly relate pad or paddle size, type or position to successful defibrillation, ROSC or long-term survival. Rather, surrogate endpoints, primarily transthoracic impedance, have been used to determine the correct pad size. Operators can use either hand-held paddles or self-adhesive pads. Multiple studies have demonstrated that paddle or pad size can alter the transthoracic impedance, which may affect the defibrillation success.[43–45] Large pads have been shown to have lower transthoracic impedance than smaller pads. The AHA currently recommends the largest paddle or pad size that can be placed on a child’s chest that should be used.[46] In small children, care must be taken to ensure that the pads do not overlap.[47]

The AHA recommends three positions for successful defibrillation. Paddles and electrode pads should be placed on the exposed chest in the anterolateral (antero-apical) position. Acceptable alternatives to this position are also the anteroposterior (paddles and pads) and the apex-posterior (pads) positions. Ideally, the goal is to position the heart between the pads so the current flow through the heart is optimized. Studies to determine the best position of pads and paddles have only been conducted in adult patients. Garcia and Kerber demonstrated that these three positions had equivalent and acceptable transthoracic impedance in transthoracic defibrillation.[48] While defibrillating a patient in an anteroposterior position, Dodd *et al*. demonstrated lower transthoracic impedance if paddles were used instead of self-adhesive pads and if the patient was placed in a left lateral position. Deakin *et al*.[49,50] established that if using a paddle in the apical position, there was a lower transthoracic impedance when the paddle was positioned longitudinally rather than horizontally.

Both self-adhesive pads and hand-held paddles are effective for defibrillation in children. The benefits of self-adhesive pads are that they are commercially available, contain the appropriate gel, may be left on the patient in proper position for re-use for a short period of time and are hands-free during delivery of the shock. An important factor is that self-adhesive pads need to be applied firmly or the transthoracic impedance can increase. The hand-held paddles require administration of a gel or paste to reduce the transthoracic impedance. This step can easily be forgotten during a crisis.

## AEDS

In 1979, portable defibrillators called AEDs were first described.[51] AEDs distinguish shockable (VT/VF) from non-shockable rhythms and recommend a shock if indicated. They are small, relatively inexpensive and can be used by minimally trained personnel. In 1995, public access programs were advocated by the AHA.[52] The Public Access Defibrillation (PAD) trial was conducted from July 2000 through September 2003, and demonstrated more survivors in the sites where the volunteers were trained in cardiopulmonary resuscitation (CPR) and AED use compared to sites with just CPR.[53] A metaanalysis found similar results.[54] Now, survival from out-of-hospital VF can be as high as 75% if defibrillation is delivered within the first 3 min.[55]

As the first AEDs were developed using adult databases of rhythms and were set to deliver a shock at 150–360 J, their use was restricted to adults. Reports of successful defibrillation in children using adult AEDs began as early as 1998.[38,56] Market clearance for pediatric attenuated electrode pad/cable system for use with adult AEDs occurred in 2001. Multiple investigators have demonstrated accurate rhythm analysis for children with high sensitivity and specificity for shockable and non-shockable rhythms.[57–59] Atkins,[29] Bar-Cohen[39] and Divekar[40] have reported successful defibrillation using the pediatric pads and cables with AED in children less than 8 years of age. In 2003, use of AEDs in children aged 1–8 years was incorporated into the CPR guideline.[60] AEDs have been successfully used in high-risk infants <1 year of age.

## SUMMARY

There is new interest in studying defibrillation in children as we now recognize that VF, although uncommon, occurs in children and is associated with a higher survival. Because much of our practice of defibrillation is derived from adult studies, questions remain about the optimal techniques for pediatric defibrillation. Data regarding energy dose in children is lacking and the current recommendations may not reflect optimal management. The role of escalating doses also remains unclear. Although research in children is difficult, the need for it is critical so that we can provide an equivalent standard of care for children in cardiac arrest and improve the outcomes.
